# Characterization and Fabrication of the CFM-JTE for 4H-SiC Power Device with High-Efficiency Protection and Increased JTE Dose Tolerance Window

**DOI:** 10.1186/s11671-020-03443-5

**Published:** 2020-11-10

**Authors:** Yi Wen, Xiao-jie Xu, Meng-ling Tao, Xiao-fei Lu, Xiao-chuan Deng, Xuan Li, Jun-tao Li, Zhi-qiang Li, Bo Zhang

**Affiliations:** 1 School of Electronic Science and Engineering, University of Electronic Science and Technology of China, Chengdu, 610054 China; 2 Microsystem and Terahertz Research Center, China Academy of Engineering Physics, Mianyang, 621900 China

**Keywords:** Silicon carbide, Ultra-high voltage, CFM-JTE, High efficiency

## Abstract

A 13.5 kV 4H-SiC PiN rectifier with a considerable active area of 0.1 cm^2^ is fabricated in this paper. Charge-field-modulated junction termination extension (CFM-JTE) has been proposed for satisfying the requirement of ultra-high reverse voltage, which enlarges the JTE dose tolerance window, making it approximately 2.8 times that of the conventional two-zone JTE. Besides, the CFM-JTE can be implemented through the conventional two-zone JTE process. The measured forward current is up to 100 A @ *V*_F_ = 5.2 V in the absence of carrier lifetime enhancement technology. The CFM-JTE structure accomplishes 96% of the theoretical breakdown voltage of the parallel plane junction with a relatively small terminal area of 400 μm, which contributes to achieving the Baliga’s figure of merit of 58.8 GW/cm^2^.

## Introduction

Silicon carbide has become a new option for improving power applications due to its characteristics of higher voltage operating at thinner active layers, extended power density, higher frequency switching, better heat dissipation, smaller system size and lower system cost [1, 2]. In the past few years, commercial SiC rectifiers and MOSFETs have been rated at 1.2–1.7 kV. However, in representative application fields such as smart grids, electric vehicles, pulse power supplies and ultra-high-voltage solid-state power supplies, there is increasing demand for blocking capability of over 10 kV and forward current capability of over 1000 A cm^−2^.

Up to now, the main challenges faced by 10 kV and higher-level SiC power electronic devices have focused on the performance of the junction termination technology, simplification of fabrication processes, high quality of thick epitaxial layer and reduction in surface defects. For ultra-high-voltage SiC devices, the length of the terminal structure is mostly 6–8 times that of the epitaxial thickness [3], which tremendously reduces the utilization rate of the wafer and thus increases the cost for fabrication. 4H-SiC PiN rectifiers have become the most suitable candidates for ultra-high-voltage applications as a result of the conductivity modulation effect. For the field limiting ring (FLR) structure, a precise optimization design can be obtained through analytical calculation [4], while the current photolithography cannot accomplish the accurate space and width of the rings. The novel field limiting ring terminal for 10 kV SiC device has been applied to have a high reverse protection efficiency [5, 6], but its terminal area exceeds 700 μm, resulting in additional consumption of the SiC wafer. Junction termination extension (JTE) is another frequently used high-efficiency terminal protection structure, but its efficiency is extremely sensitive to the dose of JTE. For ultra-high-voltage levels, MZ-JTEs and CD-JTEs [7] are utilized to critically modulate the electric field and require strict ion implantation conditions and times, which in turn increase the manufacturing complexity and cost. In order to improve the conduction ability, researches on characteristics of barrier height between diverse metals and SiC have been carried out [8, 9]. Usually, 50–100-nm-thick Ti/Al film is formed for the anode ohmic contact and Ni film is for cathode ohmic contact, respectively. Besides, the scale of the active area of the 4H-SiC rectifier will greatly affect the forward current characteristics. It is found that in the 4H-SiC N-type epitaxial layer, the *Z*_1/2_ center (*E*_C_—0.65 eV), the acceptor level of the carbon monovacancy, mainly affects the carrier lifetime [10]. Aluminum ion implantation will lead to a large concentration of massive deep levels involving the *Z*_*1/2*_ center in the mesa periphery and junction termination region [11], resulting in a decrease in carrier lifetime. Therefore, 4H-SiC rectifiers with a large active area (> 9 mm^2^) are required in design and manufacture for the impact of the reduced carrier lifetime in the mesa periphery region and termination region is relatively negligible.

In this paper, the 4H-SiC CFM-JTE PiN rectifier is fabricated on a 100-μm epitaxial layer of 5 × 10^14^ cm^−3^ and achieves considerable blocking capacity of 13.5 kV in the off state and forward current of 100A @ *V*_*F*_ = 5.2 V in the on state. The differential on-resistance of the CFM-JTE PiN rectifier is measured of 3.1 mΩ cm^2^ at room temperature. The CFM-JTE obtains 96% of the theoretical breakdown voltage through the concept and analysis of charge field modulation, which favorably expands the tolerance window of the implantation dose and leads to an acceptably termination length of 400 μm.

## Methods

### Device Structure Analysis

**Fig. 1:**
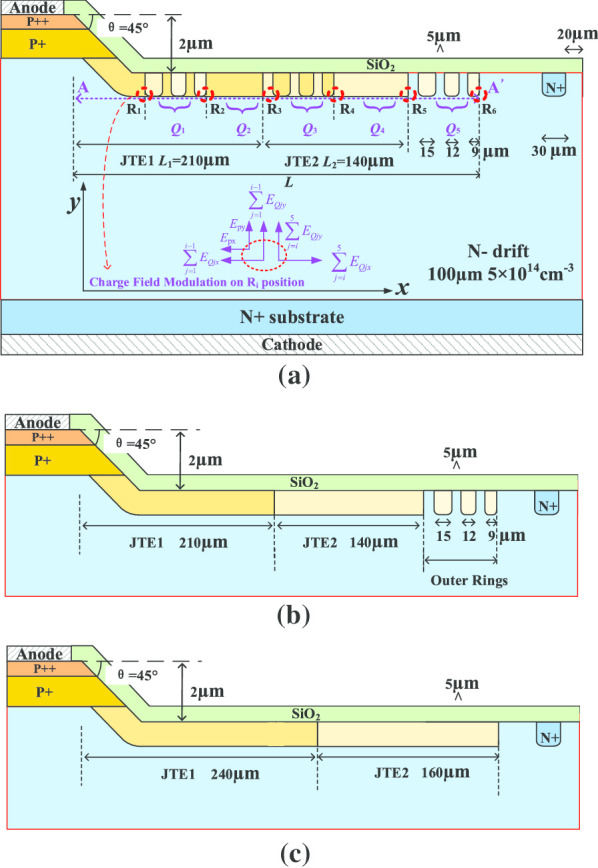
Schematic section of 4H-SiC PiN rectifier with **a** CFM-JTE, **b** ORA-JTE, **c** conventional TZ-JTE

The design, optimization and analysis are executed by Silvaco-TCAD. Figure 1 shows the schematic of the 4H-SiC PiN structure with termination, involving: (a) charge-field-modulated (CFM-JTE), (b) out-ring-assisted JTE (ORA-JTE), and (c) two-zone JTE (TZ-JTE). In the blocking state, the electron–hole collision ionization rate is closely related to the electric field strength. A concept of charge electric field modulation *E*_q_(***r***) is proposed to reveal the modulation mechanism of the CFM-JTE through the vector superposition method of the terminal electric field caused by the charge electric field *E*_q_(***r***) in Fig. 1a. The CFM-JTE consists of JTE1 region, JTE2 region and three groups of rings. The multiple rings equivalently divide the terminal into five doped zones: R_1_-R_2_, R_2_-R_3_, R_3_-R_4_, R_4_-R_5_ and R_5_-R_6_, where effective charges of *Q*_1_, *Q*_2_, *Q*_3_, *Q*_4_ and *Q*_5_ are introduced, respectively. Based on the decomposition and superposition of electric field vectors at ***x*** and ***y*** coordinates, the overall electric field located in *R*_i_ point caused by the applied potential field *E*_p_(***r***) and the charge electric field *E*_Qi_(***r***) generated by every *Q*_i_ can be analytically expressed in the ***x*** and ***y*** directions, as given in Eqs. (1) and (2), respectively.

1 $$E_{Ri, x} = E_{px} + \mathop \sum \limits_{j = 1}^{i - 1} E_{Qjx} - \mathop \sum \limits_{j = i}^{5} E_{Qjx}$$

2 $$E_{Ri,y} = E_{py} + \mathop \sum \limits_{j = 1}^{i - 1} E_{Qjy} + \mathop \sum \limits_{j = i}^{5} E_{Qjy}$$

In the off state, the low-doped depletion region contracts prematurely and aborts at the termination region due to the curvature effect in the PiN main junction. Thus, the applied potential field *E*_p_(***r***) is fulsomely concentrated at the main junction periphery. The existence of *Q*_i_ promotes the depletion along the CFM-JTE to the outermost epitaxial layer. The strength of the charge electric field *E*_Qi_ is associated with the quantity of *Q*_i_. The decrease in *Q*_i_ along the terminal outer edge effectively achieves the electric field modulation in the terminal region. Consequently, through the mechanism and effect of charge electric field modulation, CFM-JTE availably overcomes the deficiency of low diffusion coefficient of SiC to form the varied lateral doping (VLD) effect, which is a highly effective, robust and mature junction terminal protection technology for Si devices [12, 13]. The ring width (*w*_r_) of each group is decreased to 15 μm, 12 μm and 9 μm, respectively. The space of each ring is equal to the same value of 5 μm. The length and dose of the JTE1 region and the JTE2 region are both at a fixed ratio of 3:2. The detailed parameters of the proposed rectifier are given in Table 1.

**Table 1:** Structure parameters of the CFM-JTE 4H-SiC PiN rectifier

Parameter	Value
N^−^ drift doping	5 × 10^14^ cm^−3^
N^−^ epitaxial layer thickness	100 μm
N^+^ substrate doping	1 × 10^18^ cm^−3^
P^+^ layer doping	1 × 10^18^ cm^−3^
P^++^ layer doping	5 × 10^19^ cm^−3^
P^+^ layer thickness	1 μm
P^+^ layer thickness	0.5 μm
Mesa depth	2 μm
Ring space	5 μm
Ring depth	1 μm
JTE1 length/JTE2 length	3:2
Dose_JTE1/Dose_JTE2	3:2

### Simulation and Optimization

In order to decrease the deviations caused by the sensitivity of the device structure and doping concentrations, the processing configuration Athena is applied. The doping concentrations of the CFM-JTE are formed through multiple steps of aluminum implantation process simulation. The total depth of doping reaches nearly 1 μm.

**Fig. 2:**
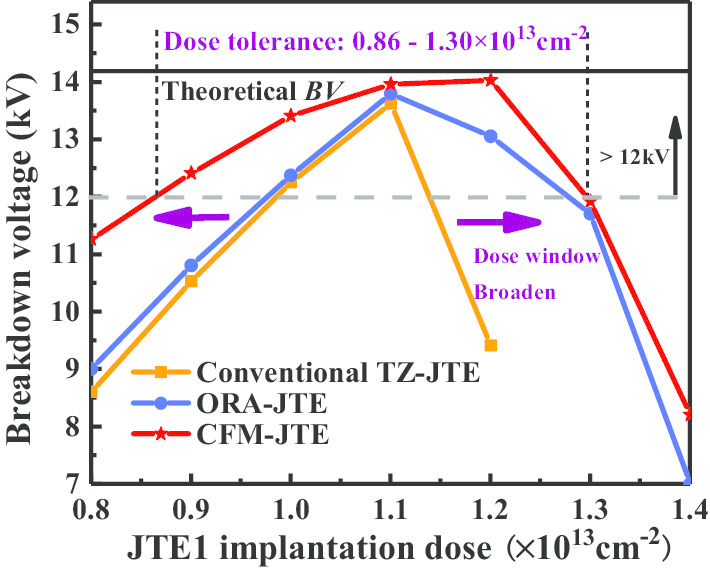
Comparison of breakdown capacities to the JTE dose window for the 4H-SiC PiN rectifier with CFM-JTE, ORA-JTE and conventional TZ-JTE

Figure 2 shows the blocking capacities and the tolerances to implantation dose of CFM-JTE, ORA-JTE and conventional TZ-JTE. The four termination structures share a fixed-length value of *L* = 400 μm to compare their efficiency. Both the length and dose of JTE1 and JTE2 are fixed at the ratio of 3:2. The simulation is performed on the 2-D structure, and the breakdown judgment criterion is that the reverse leakage current reaches 1 × 10^–9^ A. The black solid line represents the theoretical *BV* which is calculated as 14.1 kV according to [10, 14], and the gray dashed line indicates 12 kV. The design margin of 20% is applied in the target for 10 kV considering the process tolerance and simulation deviation. In the conventional TZ-JTE, the breakdown voltage is quite sensitive to the implantation dose and reaches over 12 kV only when the dose varies between 0.98 and 1.14 × 10^13^ cm^−2^. The sensitivity of ORA-JTE to JTE injection concentration is alleviated, and a wider tolerance window of 0.97–1.28 × 10^13^ cm^−2^ is obtained to maintain the target voltage above 12 kV. The CFM-JTE has the widest tolerance of the implantation dose window in a range of 0.86–1.30 × 10^13^ cm^−2^, which is approximately 2.8 times that of the conventional TZ-JTE and 1.4 times that of ORA-JTE. Thus, the CFM-JTE shows better robustness for process variations.

**Fig. 3:**
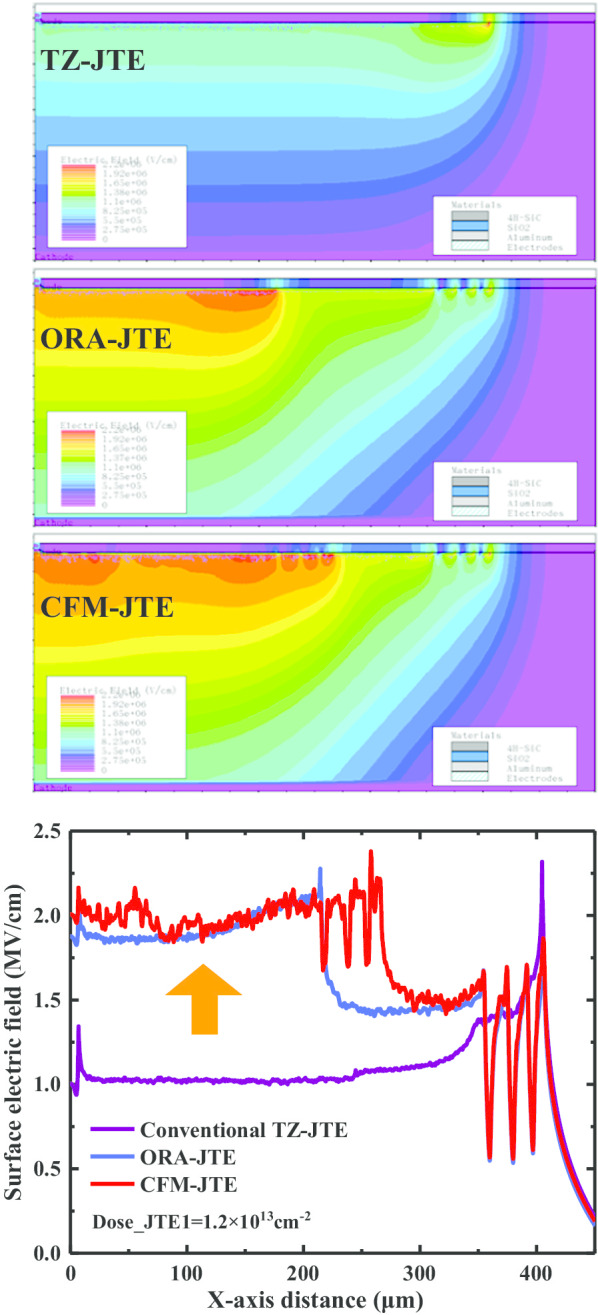
Reverse surface electric field distribution and intensity in CFM-JTE, ORA-JTE and conventional TZ-JTE

Figure 3 shows the comparison of the surface electric field distribution and intensity of CFM-JTE, ORA-JTE and TZ-JTE in the blocking state. The peak electric field is mainly concentrated in the main junction and the terminal periphery. CFM-JTE availably flattens the electric field distribution and promotes the electric field strength along the terminal, which ultimately improves the blocking capacity efficiently.

**Fig. 4:**
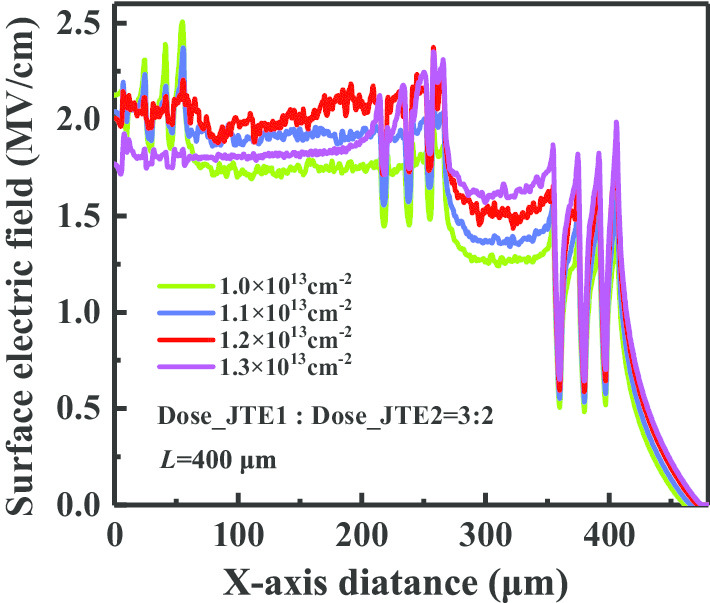
Surface electric field distributions in breakdown corresponding to different implantation doses of JTE1

In order to comprehend the mechanism of CFM-JTE modulating charge electric field, the distributions of the breakdown electric field across AA’ cutline in Fig. 1a with different implantation doses of JTE1 are plotted in Fig. 4. The existence of *Q*_i_, especially the highly doped *Q*_1_ zone next to the main junction, immensely alleviates the concentration of electric field lines at the mesa etching corner. The *Q*_5_ zone is set to ease the electric field crowding at R_5_ point in Fig. 1a. The results demonstrate that the uniformity of the electric field distribution can be effectively improved through the modulation of the charge electric field *E*_Qi_(***r***) in the terminal. Thus, the capability of the blocking voltage and the reliability of the device could be improved.

**Fig. 5:**
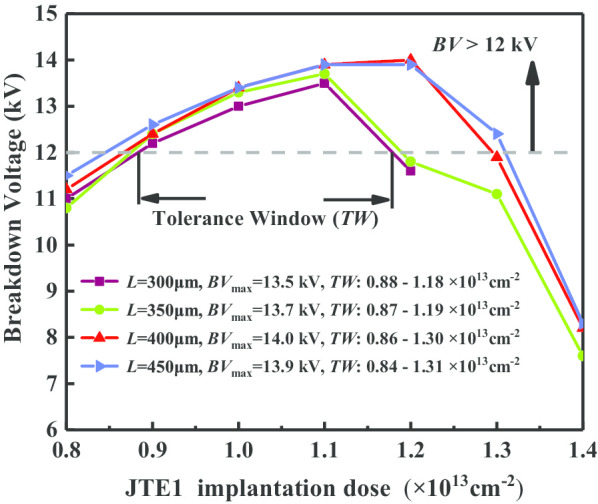
Blocking capacities with different terminal lengths

The size of terminal area affects the efficiency of the chip utilization directly. In the blocking state, the applied potential field is clustered around the main junction periphery. The JTE1 region near the main junction needs to introduce more charges to enhance the modulation effect of the charge field (*E*_q_). Thus, *L*_1_ needs to be set longer than *L*_2_. When the ratio of *L*_1_ to *L*_2_ is fixed at 3: 2, the blocking capacities of different lengths *L* on the terminal are comparatively analyzed in Fig. 5.

**Fig. 6:**
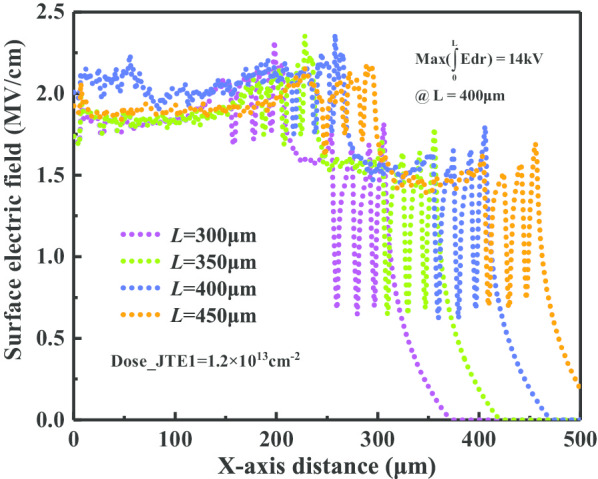
Surface electric field distributions in breakdown corresponding to different terminal lengths

The corresponding electric field distributions are displayed in Fig. 6. The large-scale area of CFM-JTE is conducive to increasing the required charge *Q*_i_ and giving full play to the modulation effect of *E*_Qi_(***r***), so as to obtain a larger implantation dose tolerance window (TW). When the CFM-JTE length *L* is set as 300 μm, 350 μm, 400 μm and 450 μm, the TW range increases sequentially, corresponding to 3 × 10^12^ cm^−2^, 3.2 × 10^12^ cm^−2^, 4.4 × 10^12^ cm^−2^ and 4.7 × 10^12^ cm^−2^ in range on condition that the BV is over 12 kV. It is more appropriate to select a terminal length *L* of 400 μm in this work based on the trade-off of terminal size, JTE dose tolerance window, terminal electric field modulation and breakdown voltage capability.

**Fig. 7:**
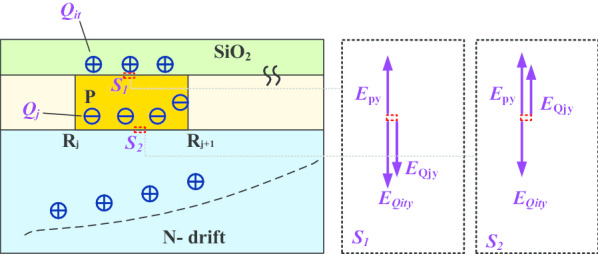
Electric field modulation at *S*_1_ and *S*_2_ by interface positive charges (*Q*_it_), effective charges (*Q*_j_) and applied potential

The existence of surface states should be considered in the actual fabrication of the 4H-SiC PiN rectifier. These surface states are derived from holes trapped in deep interface states, fixed oxide charges of P-SiC (including P^++^ layer and P-JTE region)/SiO_2_ interface and the implementation process [15–18]. For the CFM-JTE termination, the electric field modulation at the interface *S*_*1*_ and *S*_*2*_ by interface positive charges (*Q*_it_), effective charges (*Q*_j_) and applied potential in the direction of the vertical is analyzed in Fig. 7.

**Fig. 8:**
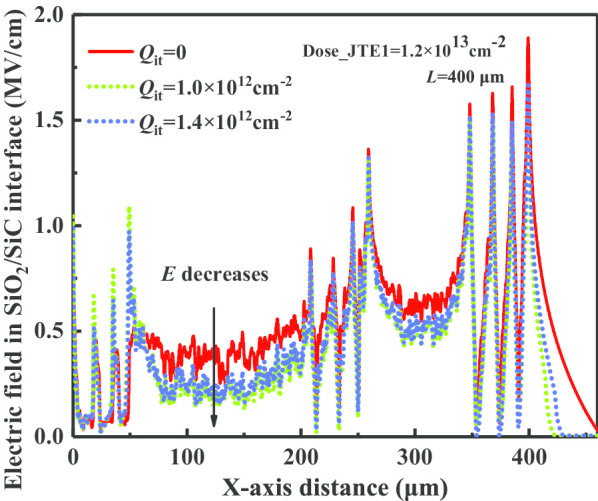
Electric field distributions in SiO_2_/SiC interface with different *Q*_it_

Positive interface charges (*Q*_it_) generate an opposite charge field (*E*_Qity_) compared with the applied potential field (*E*_py_), helping to mitigate the electric field strength in the direction of the vertical interface. Figure 8 exhibits the electric field distributions in SiO_2_/SiC interface *S*_*1*_ under conditions of different *Q*_it_.

**Fig. 9:**
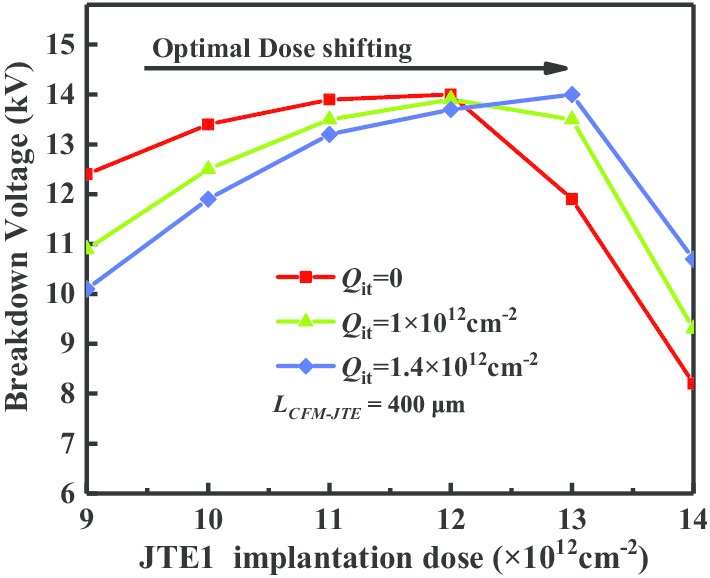
Comparison of influences to the blocking capacity by interface charges in 4H-SiC PiN rectifier

In terms of the charge field modulation analysis at interface *S*_*2*_, *E*_Qity_ is opposite to the vector direction of *E*_py_ and *E*_Qjy_. The existence of *Q*_it_ helps reduce the electric field in *S*2. Considering the existence of the interface charge (*Q*_it_), a larger ionization effective charge *Q*_j_ is required to counteract the electric field intensity generated by the *Q*_it_. Consequently, when the quantity of *Q*_it_ increases, the optimal implantation dose concentration of JTE should be simultaneously enhanced to maintain the same blocking capability. As shown in Fig. 9, the overall *BV–Dose* curve shifts in the direction of rising with the increase in *Q*_it_.

## Results and Discussion

**Fig. 10:**
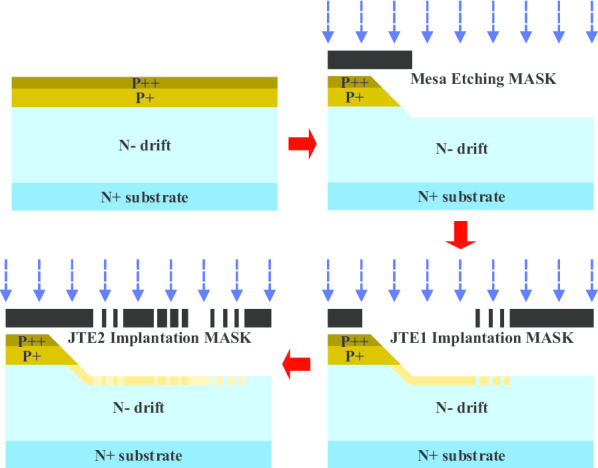
Process flow of the CFM-JTE

The proposed CFM-JTE 4H-SiC PiN rectifier has been manufactured in a 4-inch N-type wafer with 4° off-axis (0001). The wafer is grown through epitaxy with four layers (N^+^, N^−^, P^+^, P^++^) corresponding to the concentration of 1 × 10^18^ cm^−3^, 5 × 10^14^ cm^−3^, 1 × 10^18^ cm^−3^ and 5 × 10^19^ cm^−3^. The major process flow of the CFM-JTE fabrication is displayed in Fig. 10. The P^++^ layer is grown through epitaxy to obtain uniform and highly doping to promote the ohmic contact quality between SiC and anode metal. The mesa etching structure is formed in the ICP-RIE facility through the mesa-etching mask. The etching gas is mainly composed of SF_6_ and oxygen. Multiple Al ion implantations are implemented at a maximum energy of 500 keV to form the CFM-JTE structure. A two-step Al ion implantation is applied to form the five diminishing doped zones. JTE1 and R_2_-R_3_ zones are formed through the first implantation mask. JTE2, R_1_-R_2_, R_3_-R_4_, R_4_-R_5_ and R_5_-R_6_ regions are configured simultaneously by the Al ion implantation through the second implantation mask. As is known to all, the ion activation rate in silicon carbide is not high after implanted with high-energy ions, accompanied by severe lattice damage. In order to improve the above undesirable situation, the aluminum ion implantations are accomplished at a temperature of 500 °C through an oxide mask. Post-implantation annealing has been conducted in argon ambient at a temperature of 1800 °C for 10 min with a carbon cap in order to further renovate the damage caused by high-energy ion implantation and improve the accuracy of doping concentration by increasing the effective ion activation rate. P-type SiC ohmic contact is formed using Al/Ti. The RTA process is consistently carried out and inspected for two minutes in an inert gas nitrogen environment at a temperature of 1000 °C. High-quality passivation layers (SiO_2_ layer, Si_3_N_4_ film and thick polyimide layer) are deposited to prevent surface leakage and avoid sparking in air [5]. The CFM-JTE PiN rectifier covers an active area of up to 0.1 cm^2^. The fabrication process is consistent with the conventional two-zone JTE 4H-SiC PiN rectifier, without any extra masks or process steps, which is extremely conducive to reduce the manufacturing complexity and cost.

**Fig. 11:**
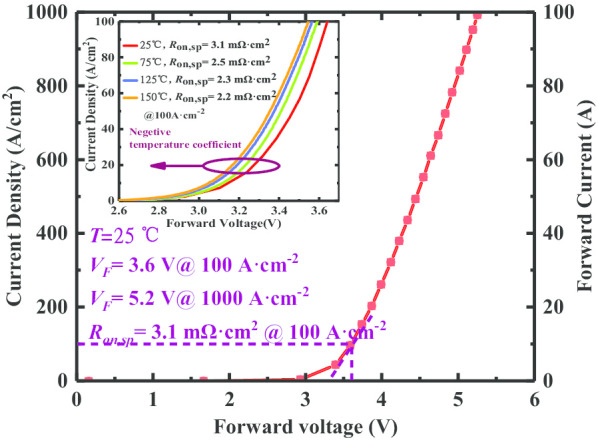
Forward *I–V* curve of the CFM-JTE PiN rectifier and characteristics at diverse temperature environments in the inset

The forward characteristics of the manufactured CFM-JTE PiN rectifier are tested on the wafer using the CS-3200 Curve Tracer instrument. The fabricated CFM-JTE PiN rectifier exhibits a large capacity of forward current conduction without carrier lifetime enhancement technology. The forward current is measured up to 100 A corresponding to a forward voltage of 5.2 V, as shown in Fig. 11. The differential on-resistance of the proposed rectifier is measured as 3.1 mΩ cm^2^ at room temperature, corresponding to the forward voltage of 3.6 V. The forward conduction characteristics at different temperatures are also illustrated in the inset of Fig. 11. The *I–V* curve shows negative temperature coefficient peculiarity. This is because as the temperature rises, the mobility of the material decreases, while the narrower band gap of the SiC material reduces the self-built potential of the P–N junction, and the carrier lifetime of the drift region extends with the increase in temperature, thereby leading to an increase in the current density. The measured turn-on voltage is defined at a forward current density of 10 A·cm^−2^. It gradually reduces from 3.14 to 3.04 V when the ambient temperature rises from 25 to 150 °C. The maximum shift of the forward turn-on voltage has been stuck in a range of 3%, corresponding to a value of 0.1 V, which exhibits much better temperature stability than Si PiN.

**Fig. 12:**
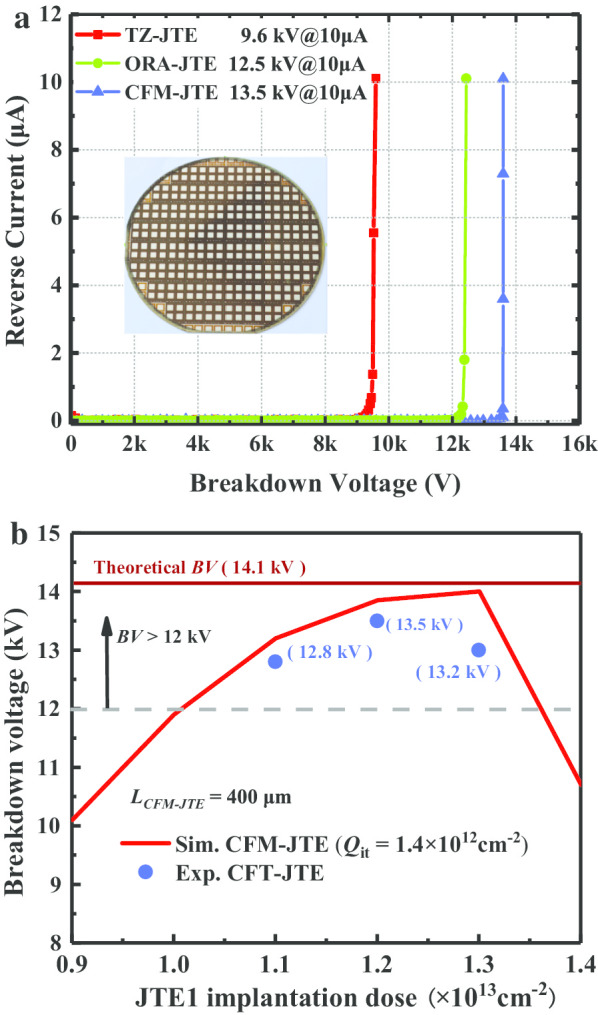
Breakdown characteristics of the fabricated 4H-SiC PiN rectifier. **a** Measured *BV* of the CFM-JTE, ORA-JTE and TZ-JTE. **b** Measured BV of the CFM-JTE with different implantation dose. Solid marks are the experimental values

The 4H-SiC PiN rectifiers with CFM-JTE, ORA-JTE as well as conventional TZ-JTE are fabricated on the 4-inch wafer, and their terminal protection effects are shown in Fig. 12. Reverse breakdown voltage measurements are executed and immersed in Fluorinert oil to avoid arcing in the air. In experimental measurements with JTE1 dose of 1.2 × 10^13^ cm^−2^, the PiN rectifiers with ORA-JTE and the conventional TZ-JTE obtain the blocking capacities of 12.5 kV and 9.6 kV, respectively, with the same terminal length of 400 μm.

Multi-batch samples of CFM-JTE PiN rectifiers could repeatedly obtain the breakdown voltage of 13.5 kV at the leakage current of 10 μA. The experimental blocking voltage reaches up to 96% of the theoretical breakdown value, which indicates that the CFM-JTE has a higher terminal protection efficiency. A withstand voltage of more than 130 V/μm has been achieved in the 100 μm drift layer of the 4H-SiC PiN rectifier. The Baliga’s figure of merit (BFOM = BV^2^/*R*_on,sp_) reaches 58.8 GW/cm^2^ at room temperature. The CFM-JTE PiNs with different JTE1 implantation doses have been manufactured. The experimental and simulated values of the breakdown voltage are depicted in Fig. 12b. The experimental values are in accordance with the trend of simulation and confirm that the CFM-JTE structure can expand the JTE dose tolerance window effectively. Table 2 compares the characteristics of the recently reported ultra-high-voltage 4H-SiC rectifiers. The CFM-JTE 4H-SiC rectifier fabricated in this work shows excellent performance in the aspects of ultra-high-voltage blocking capacity, ultra-high forward current conduction capacity and high termination efficiency.

**Table 2:** Summary and comparison of characteristics of the recently reported 4H-SiC PiN rectifiers and this work

References	BV (kV)	Thickness (μm)	Active area (mm^2^)	Current density (A/cm^2^) @ *V*_*F*_ (V)	*V*_*F*_ (V) @ 100 A/cm^2^	*R*_on,sp_ (mΩ·cm^2^) @ *V*_*F*_ (V)	Termination length (μm)	Termination efficiency (%)
This work	13.5	100	10	1000 @ 5.2	3.6	3.1 @ 3.6	400	96
[19]	27.5	239	5.75	300 @ 20	16	28 @ 7.6	–	83
[20]	10	150	9	222 @ 5.2	4.8	3 @ 12.5	400	50
[21]	10.2	120	< 0.5	–	–		450	88
[22]	15	147	< 0.5	100 @ 9.7	9.7	62 @ 9.68	500	93
[23]	13	98	–	120 @ 3.3	3.2	1.87 @ 3.2	–	81

## Conclusions

In this work, the 4H-SiC CFM-JTE PiN rectifier has been designed and fabricated successfully. The experimental breakdown voltage of the CFM-JTE PiN is 13.5 kV corresponding to 96% of the theoretical blocking value. The CFM-JTE exhibits a much improved terminal protection efficiency compared with the ORA-JTE (BV = 12.5 kV, with protection efficiency up to 88%) and the conventional TZ-JTE (BV = 9.6 kV, with protection efficiency up to 68%). The CFM-JTE PiN rectifier acquires a large forward current of 100 A @ *V*_F_ = 5.2 V without using carrier lifetime enhancement technology and exhibits stable forward *I–V* characteristics at various temperatures. The Baliga’s figure of merit for the CFM-JTE PiN rectifier reaches 58.8 GW/cm^2^ at room temperature. The tolerance of the implantation dose window for CFM-JTE is enlarged based on the charge electric field modulation, which is approximately 2.8 times that of the conventional TZ-JTE and 1.4 times that of ORA-JTE, showing much better robustness to process variation. In addition, the manufacturing process for CFM-JTE is consistent with the conventional TZ JTE process without increasing the number of exceptionally complex processes or masks, which demonstrates that the CFM-JTE is optimal for ultra-high-power applications with satisfactory terminal efficiency and process tolerance.

## Data Availability

All the data are available without restrictions.

## Abbreviations

SiC: Silicon carbide
JTE: Junction termination extension
CFM: Charge field modulated
TZ-JTE: Two-zone junction termination extension
FLR: Field limiting ring
MZ-JTE: Multiple-zone junction termination extension
CD-JTE: Counter-doped junction termination extension
Ti: Titanium
Al: Aluminum
*E*_c_: Energy conduction band
*V*_*F*_: Forward voltage
ORA-JTE: Out-ring-assisted junction termination extension
*E*_q_: Charge electric field
*E*_p_: Applied potential field
*Q*_i_: Effective charge
VLD: Varied lateral doping
*w*_r_: Ring width
2D: Two dimensional
BV: Breakdown voltage
TW: Tolerance window
*Q*_it_: Interface charge
*Q*_j_: Ionization effective charge
RTA: Rapid thermal annealing
*R*_on,sp_: Specific on-resistance
BFOM: Baliga’s figure of merit
